# EXPLORING REFERRAL PATTERNS AND PATIENT PROFILES IN A CLINICAL EXERCISE PROGRAM FOR OLDER ADULTS

**DOI:** 10.1093/geroni/igae098.2451

**Published:** 2024-12-31

**Authors:** Stephen Jennings, Jamie Giffuni, Neil Gregor, Tyara Mason, Marc Pepin, Katherine Hall

**Affiliations:** Durham Veterans Affairs Healthcare Adminstration, Durham, North Carolina, United States; Baltimore and Loch Raven VAMC, Baltimore, Maryland, United States; Veterans Health Administration, Martinez, California, United States; Durham VA Health Care System, Durham, North Carolina, United States; Durham VA Health Care System, Durham, North Carolina, United States; Veterans Health Administration/Duke University, Durham, North Carolina, United States

## Abstract

Gerofit is a supervised, outpatient clinical exercise program offered to Veterans aged ≥65 enrolled in Veterans Health Administration. Enrollment in the program is contingent on health care provider referral. This analysis seeks to examine the sources and characteristics of referrals, aiming to inform strategies to enhance accessibility and optimize program efficiency. Referrals (N=2511) to the program from 2014-24 across 3 of the largest Gerofit sites were assessed, and came from a multitude of providers (primary care-44%; nutrition & dietetics-14%; physical therapy-11%; mental health-5%; etc.). Referrals from specialty clinics and physical medicine & rehabilitation physicians had the lowest enrollment rate (22% and 10%, respectively), while referrals from physical therapy and nutrition & dietetics had the highest enrollment rate (51% and 46%, respectively). Compared to all Veterans enrolled at these sites during this timeframe, those referred by nutrition & dietetics and mental health tended to be younger and have better physical performance, while specialty clinics & geriatrics tended to refer older and more frail adults. Examination of participation rates at 1 year post-enrollment by referring provider type showed that nutrition & dietetics referrals had the highest 1-year attrition levels (21%). Increased recruitment from high performing providers, and education to providers on program eligibility targeting those with lower enrollment rates (< 25%), will result in improved staff efficiency and enhance timely enrollment of eligible Veterans by reducing bottlenecks in the referral stage.

